# Using System Identification to Construct an Inherent Model of Pupillary Light Reflex to Explore Diabetic Neuropathy

**DOI:** 10.3390/brainsci11070852

**Published:** 2021-06-25

**Authors:** Yung-Jhe Yan, Chien-Nan Chen, Mang Ou-Yang

**Affiliations:** 1 Institute of Electrical and Control Engineering, National Yang Ming Chiao Tung University, Hsinchu 300, Taiwan; jerryyan.eed02g@nctu.edu.tw; 2 Institute of Biomedical Engineering, National Yang Ming Chiao Tung University, Hsinchu 300, Taiwan; t27713245110@gmail.com

**Keywords:** system identification, pupillary light reflex, autonomic nervous system, diabetic autonomic neuropathy, biological system modeling

## Abstract

This study proposed a pupillary light reflex (PLR) inherent model based on the system identification method to demonstrate the dynamic physiological mechanism of the PLR, in which pupillary constriction and dilation are controlled by the sympathetic and parasympathetic nervous system. This model was constructed and verified by comparing the simulated and predicted PLR response with that of healthy participants. The least root-mean-square error (RMSE) of simulated PLR response was less than 0.7% when stimulus duration was under 3 ms. The RMSE of predicted PLR response increased by approximately 6.76%/s from the stimulus duration of 1 ms to 3 s, when the model directly used the parameters extracted from the PLR at the stimulus duration of 10 ms. When model parameters were derived from the regression by the measured PLR response, the RMSE kept under 8.5%. The model was applied to explore the PLR abnormalities of the people with Diabetic Mellitus (DM) by extracting the model parameters from 42 people with DM and comparing these parameters with those of 42 healthy participants. The parameter in the first-order term of the elastic force of the participants with DM was significantly lower than that of the healthy participants (*p* < 0.05). The sympathetic force and sympathetic action delay of the participants with DM were significantly larger (*p* < 0.05) and longer (*p* < 0.0001) than that of the healthy ones, respectively. The reason might be that the sympathetic nervous system, which controls the dilator muscle, degenerated in diabetic patients.

## 1. Introduction

The autonomic nervous system (ANS) is composed of sympathetic nervous systems (SNS) and parasympathetic nervous systems (PSNS) [1]. SNS and PSNS antagonize each other to maintain the balance of the human body. Once ANS is out of balance, it is called dysautonomia, which deeply affects many organizations and body function, including body temperature, blood pressure, heart rate, digestion, urination, bowel movement, and pupillary light reflex (PLR). ANS dysregulation directly affects the central nervous system and organs controlled by ANS, and has apparent symptoms in passive organs [2,3]. Pupillary control involves the different neuroanatomical pathways that are mainly controlled by ANS. Thus, the abnormalities of ANS may be directly observed from PLR.

In recent years, many studies [4,5,6,7,8,9,10,11,12,13,14,15,16,17,18,19,20,21,22,23,24,25] have tried to examine the abnormalities of ANS through PLR since PLR can be quickly measured in a non-invasive way and easily be quantized for analysis by recoding pupillary images. Surakka et al. [6] explored the PLR in people with multiple sclerosis (MS) and suggested that SNS and PSNS are disturbed in people with early MS. Chougule et al. [8] concluded that abnormal re-dilation velocity of people with Alzheimer’s Disease was the most consistent result in most studies. Narita et al. [12] found that the patients with neuronopathic Gaucher disease exhibited abnormal PLR under the red-light stimulus. Moreover, some studies explored the abnormalities of PLR in people with diabetic autonomic neuropathy by calculating the indices relating to the amplitude, duration, and velocity of pupillary constriction and dilation [13,14,15,16,17,18,19,20,21,22,23,24,25]; resting pupil diameter of people with diabetes mellitus (DM) was significantly smaller than that of healthy people [13,14,15,16,17,18,19,20,21,22,23,24]; minimum pupil diameter (MPD) of people with DM was smaller than that of healthy people [16,17,18,19,21,24]; and reflex amplitude significantly decreased in people with DM [13,16,17,18,19,20,25]. In a previous study [24], we used a self-designed pupilometer to collect the PLR of people with and without early DM under the condition of short-pulse light stimulus; a total of 16 indices, relating to the amplitude, duration, and velocity of pupillary constriction and dilation, were used to explore the PLR abnormalities in DM; and the duration that pupil restores from its minimum size to half of its resting size (DRP), maximum pupil restoration velocity (MRV), and average restoration velocity (ARV) were significantly decreased in people with DM. The amplitude, duration, and velocity of pupillary constriction and dilation in PLR may be used to explore the severity of diabetic autonomic neuropathy but may not directly offer the relation between ANS and PLR abnormalities. However, this relation may be explored through a physiologically-based PLR model.

The earlier works of the pupillary model were based on first-order linear or seconds order nonlinear transfer function [26,27,28,29,30,31]. Privitera et al. [31] created a binocular pupil model composed of two retinal afferent pathways, (1) the mesencephalic ocular motor complex, and (2) the two oculomotor 2I nerve efferent pathways. This model completely described the neural pathway and considered the direct current and alternating current effect of the input light. However, the model is challenging to present in closed-form equations and may be too complex to use for exploring the abnormalities of ANS. Pamplona et al. [32] proposed a pupillary model that integrated the model of Moon et al. [33] and Longtin et al. [34,35]. This model was a black-box model, which mainly described PLR based on experimental data and had inconsistent fitting results with short-flash light experimental data. Usui et al. [36] proposed an inverse dynamic PLR model based on the property of the pupillary muscle represented as an elastic element, a viscous element, and an active contractile element. The model can be used to estimate the input of ANS; however, this model is too complicated in 19 equations. Fan et al. proposed a PLR model that consisted of passive muscle elastic force, viscous resistance, and the active forces generated from the ANS modulation [37]. This model is a gray box model, which can observe the works of ANS. However, the fitting error of this model might increase as the stimulus duration increase.

In this study, we proposed a PLR inherent model modified by Fan’s model [37]. To enhance the model fitting accuracy, the first-order term with the elastic constant of muscle elastic force was added in the current model. The elastic force, the viscous force, and the ANS forces were assumed to be zero when the pupil was in a dark-adapted resting state, and the SNS force was assumed to join the pupillary constriction before the maximum pupil constriction and still active in pupillary dilation while the force of PSNS stopped. This model, which used the second-order differential equation, describes the pupillary constriction and dilation innervated by SNS and PSNS. Thus, the model can extract the relative input amplitude and timing of SNS and PSNS from the measured PLR response. The PLR difference would be examined between people without and with DM. The model parameters were extracted and compared from PLR data of 42 healthy participants and 42 ones with DM.

## 2. System Identification of Pupillary Light Reflex

**Figure 1:**
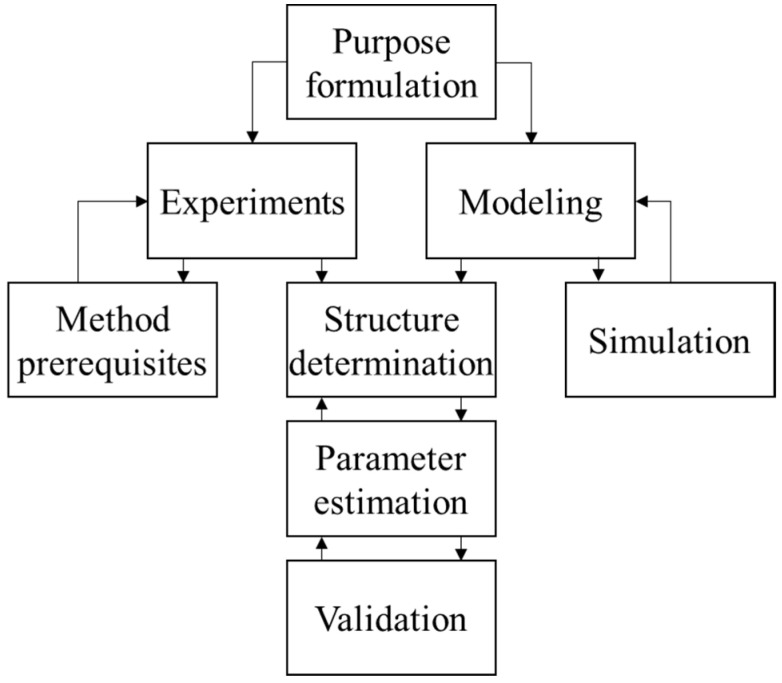
Procedures of system identification.

System identification has been an active research field for more than fifty years. It is a method of exploring an unknown system. Furthermore, the model constructed from the system identification is used to predict the behavior of the system. System identification can be divided into three main parts: experiment, modeling, and structure determination. Figure 1 depicts the architecture of the system identification theory. The model can be divided into white-box models, black-box models, and gray-box models. White box models are derived by first principles such as physical, chemical, and biological laws. Black-box models are fully based on measurement data. Gray box models combine both white-box models and black-box models. Some parameters in this model are uncertain and can be estimated by system identification [38].

### 2.1. Pupillary Light Reflex Inherent Model Construction

In this study, a physiologically-based PLR inherent model was proposed to identify the viscoelastic properties of the iris muscle and ANS input induced by the pulse of light stimuli. Thus, the force that controls the movement of the iris is combined with passive muscle elastic force, viscous resistance, and the effective force originated from ANS [37]. The force equation is written as:

(1) $$\frac{d^{2}r}{dt^{2}}=k_{d2}{\left(l_{0}-r\right)}^{2}+k_{d1}\left(l_{0}-r\right)-D\frac{dr}{dt}-F_{n}\left(t\right).$$

Equation (1) has been divided by the iris muscle mass. *r* represents the pupil radius. The passive muscle elastic force was defined as a second-order equation that was based on experimental observation [35]. $l_{o}$ is the steady-state pupil diameter in dark adaption. $k_{d2}$ is second-order elastic constants. To enhance the model fitting accuracy, the first-order term with elastic constant $k_{d1}$ of muscle elastic force equation was added in current model. D is viscous constant.

$F_{n}\left(t\right)$ is the effective muscle force, which is combined with the force *F_p_*(*t*)** from PSNS and force *F_s_*(*t*)** from SNS and expressed as:

(2) $$F_{n}\left(t\right)=F_{p}\left(t\right)-F_{s}\left(t\right),$$

where the muscle force *F_p_*(*t*) and *F_s_*(*t*) were assumed to be square-wave pulses because the muscle activation is triggered by the impulse signals from efferent nerves. *F_p_*(*t*) and *F_s_*(*t*) are written as (3) and (4), respectively.

(3) $$F_{p}\left(t\right)=f_{p0}*\left[u\left(t-\tau_{p1}\right)-u\left(t-t_{d}-\tau_{p2}\right)\right].$$

(4) $$F_{s}\left(t\right)=f_{s0}*\left[u\left(t-\tau_{s1}\right)-u\left(t-t_{d}-\tau_{p2}\right)\right]+f_{s1}*\left[u\left(t-t_{d}-\tau_{p2}\right)-u\left(t-t_{d}-\tau_{s2}\right)\right]$$

**Figure 2:**
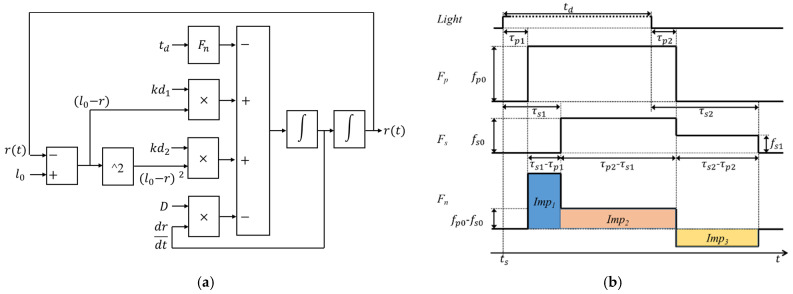
(**a**) Control block diagram of the pupillary light reflex inherent model and (**b**) timing diagram of the force originated from autonomic nervous system before and after a light stimulus.

$t_{d}$ is the duration of the light stimulus. $f_{p0}$ is force intensity originated from PSNS. $\tau_{p1}$ is the delay between the start of the light stimulus and the activation of the force $F_{p}$, and $\tau_{p2}$ is the delay between the end of the lights stimulus and the end of the force $F_{p}$. *F_n_*(*t*) was assumed to be 0 when the pupil is in a steady-state. Two square-waves ($f_{s0}$ and $f_{s1}$) were used to describe the force $F_{s}$, because this study assumed that the $F_{s}$ still act in lower force intensity $f_{s1}$, compared to $f_{s0}$, after the end of the force $F_{p}$. $\tau_{s1}$ is the delay between the start of the light stimulus and the activation of the force $F_{s}$, and $\tau_{s2}$ is the delay between the end of the light stimulus and the end of the force $F_{s}$. Figure 2 depicts the control block diagram of the PLR inherent model and how the force *F_n_* works before and after a light stimulus. Moreover, *Imp_*1*_*, *Imp_*2*_*, and *Imp_*3*_* were signs that PSNS dominated the movement of iris; PSNS and SNS antagonized each other during the movement of the iris, and SNS dominated the movement of the iris, respectively. These parameters were mainly used to compare the difference between people with and without DM.

### 2.2. Pupillary Light Reflex Response Experiment

The PLR response was collected by the customized pupilometer [24]. The pupilometer stimulated the right eye of healthy participants and captured the images of both eyes for 5 s before the stimulation and 10 s afterward. The participants took a dark-adaption in a dark room for 2 min and had seven tests in a dark room afterward. The stimulus duration of seven tests was 1ms, 10 ms, 100 ms, 500 ms, 1 s, 2 s, and 3 s, and the stimulus lights of seven tests were all 0.12 cd white light. The participants rested for 15 min between the tests.

The PLR response was captured in sequential still images. The pupil radius was extracted from each image using MATLAB (The MathWorks, Portola Valley, CA, USA). The complete procedure of extracting pupil radius was illustrated in a previous study [24]. Briefly, the procedure was divided into several steps as follows: increasing contract of image, binarization, edge detection, bad data exclusion, and data interpolation.

### 2.3. Structure Determination and Parameter Estimation of PLR Inherent Model

The root-mean-square percentage error (RMSPE) was adopted to evaluate the goodness of simulation results of the PLR inherent model (1) and defined as:

(5) $$\mathrm{RMSPE}=\sqrt{\frac{1}{N-1}\overset{N}{\underset{i}{\sum }}{\left(\frac{X_{i}-Y_{i}}{X_{i}}\right)}^{2}},$$

where *X* is the measured PLR response data and *Y* is the model simulated PLR response.

**Figure 3:**
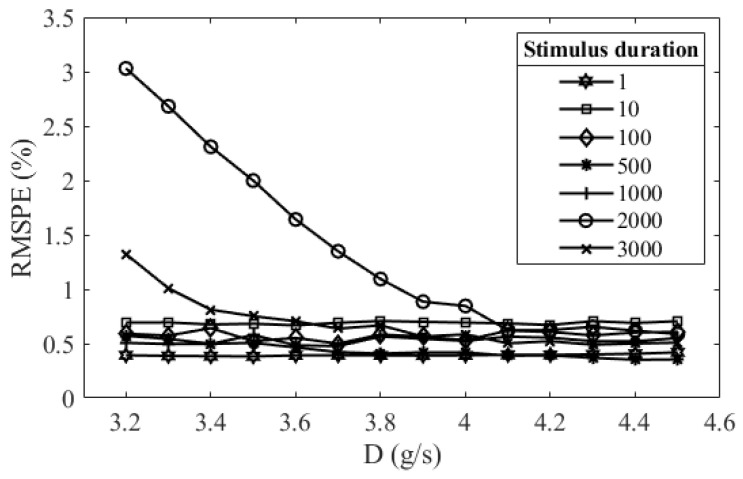
Relations between the root-mean-square percentage error (RMSPE) of model simulation results and the viscous constants when the stimulus durations were 1ms, 10 ms, 100 ms, 500 ms, 1 s, 2 s, and 3 s.

The simulation first decided the initial value and boundary condition of the parameters in the PLR inherent model for parameter searching. The initial value of the parameters was determined by applying a random search method. This method randomly generates sets of the parameters and finds a set of parameters that make the simulated PLR response have the lowest RMSPE at the stimulus duration of 500 ms. The reason for using the PLR response at this stimulus duration is that the parameters in the PLR of longer stimulus duration have lower variability. The boundary of the parameters was set to be approximately ±20% of its initial value. The RMSPEs between the model output and the measured PLR response in stimulus durations from 1 ms to 3 s were found to be approximately stable when the viscous constant D was approximately 4.3 g/s (Figure 3); therefore, the D was the constant of 4.3 g/s in all simulations and predictions. The initial value and the boundary value of the parameters are listed in Table 1.

**Figure 4:**
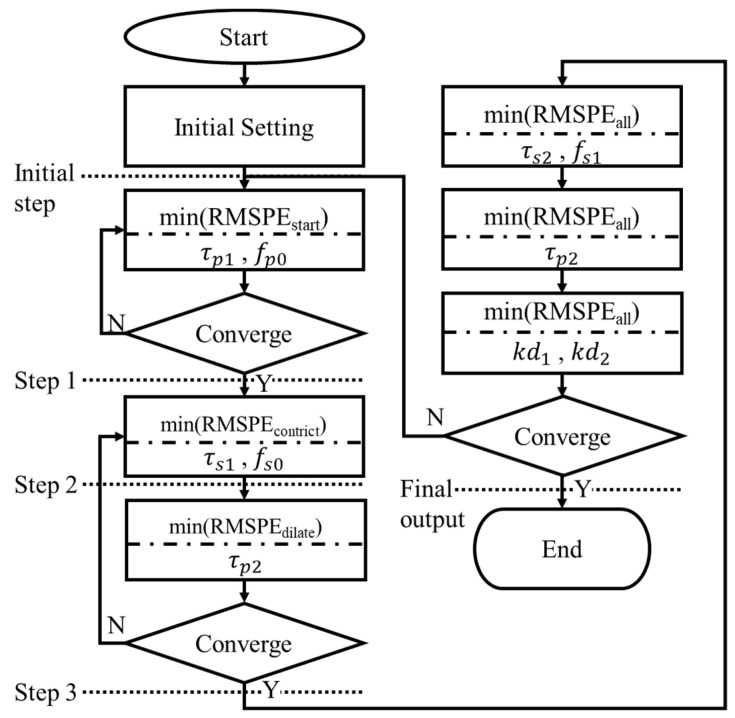
Flow chart of parameter estimation order in the model simulation.

The model simulation was based on a univariate search method that searches one parameter at a time. All parameters were searched within the interval specified in Table 1. The search of one parameter was stopped by finding the minimum RMSPE between model output and measured PLR response at a specified time interval. The convergence criterion in the simulation process was whether the searching values of the first parameter are equal before and after the second parameter is searched. The order of searching parameter, which was related to the activating and deactivating order of SNS and PSNS, is illustrated in Figure 4. The parameters related to PSNS were searched firstly; those related to SNS were searched afterward; and the parameters independent of stimulus input were searched lastly.

**Figure 5:**
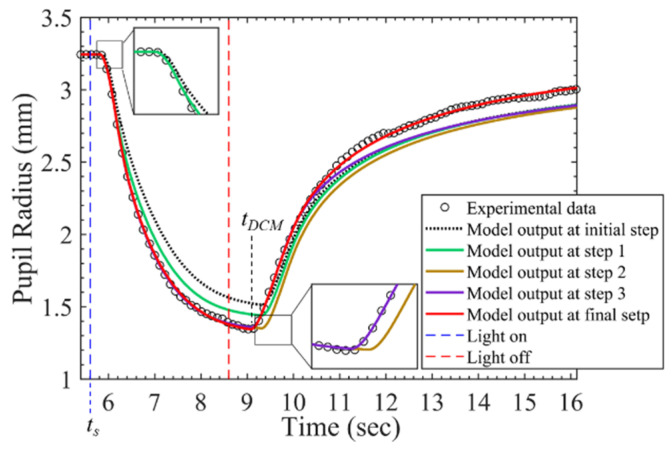
PLR inherent model output at different stages during parameter searching process.

After a pupil starts to be stimulated by a light source for hundreds of milliseconds, it starts to constrict. It was assumed that this process is dominated by only PSNS and significantly related to $\tau_{p1}$ and $f_{p0}$; thus, these two parameters were searched firstly, and the RMSPE_start_ presenting the RMSPE on the time interval from the start of stimulus *t_s_* to *t_s_* + 0.4 s was calculated. SNS starts to originate the force *F_n_* with PSNS after PSNS starts to activate by hundreds of milliseconds; *τ*_*s*1_ and *f*_*s*0_ were assumed to dominate this process and were searched secondly; the RMSPE_constrict_ presenting the RMSPE on the time interval from *t_s_* to the time t_DCM_ when a pupil constricts to its minimum size was calculated. After the pupil constricts to its minimum diameter, the pupil starts to dilate; $\tau_{p2}$, which was assumed to decide the start of dilating, was searched thirdly, and the RMSPE_dialate_ presenting the RMSPE on the time interval from *t_s_* to *t_s_* + 1.25 * (t_DCM_ − *t_s_*) was calculated. $\tau_{f2}$ and $f_{s1}$ were assumed to dominate the process during the pupil constriction and were searched fourthly; the RMSPE_all_ presenting the RMSPE on the time interval from *t_s_* to *t_s_* + 3 * (t_DCM_ − *t_s_*) was calculated. After that, $\tau_{p2}$ was searched again and *k*_*d*1_ and *k*_*d*2_ searched afterward. Figure 5 shows the model output at the initial step, step 1, step 2, step 3, and final step. The model outputs gradually close to the beginning partition of the measured PLR response at step 1, the constricting partition of that at step 2, the dilating partition of that at step 3, and overall at the final step.

**Figure 6:**
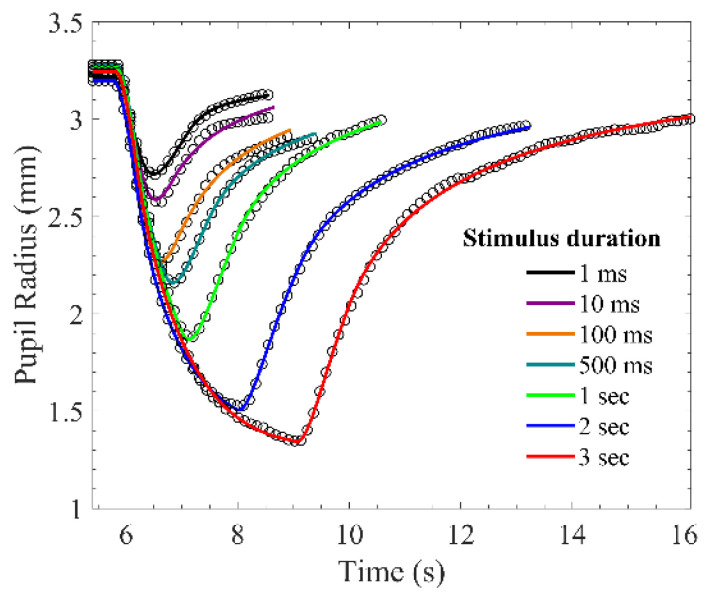
Measured pupillary light reflex (PLR) response and simulated PLR response. The circle markers are measured PLR response, and the solid lines are simulated PLR response.

**Figure 7:**
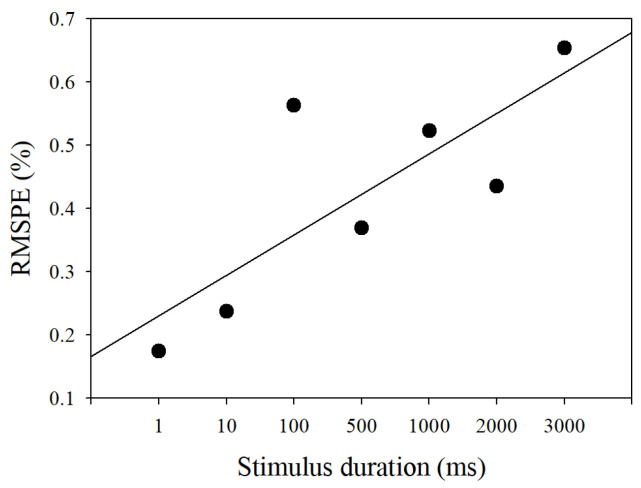
RMSPE of the simulated pupillary light reflex response under seven stimulus durations.

The model outputs with the stimulus durations from 1 millisecond to three seconds comparing to experimental PLR data are shown in Figure 6. These output curves were extremely close to those of measured PLR response. The RMSPEs between model outputs and measured PLR response in seven stimulus durations are depicted in Figure 7. Although the RMSPE with long stimulus duration was larger than the short stimulus duration, the RMSPEs were all under 0.7%. Consequently, the PLR inherent model can adequately express dark-adapted PLR under the stimulus durations from 1 ms to 3 s.

### 2.4. Validation of PLR Inherent Model

**Figure 8:**
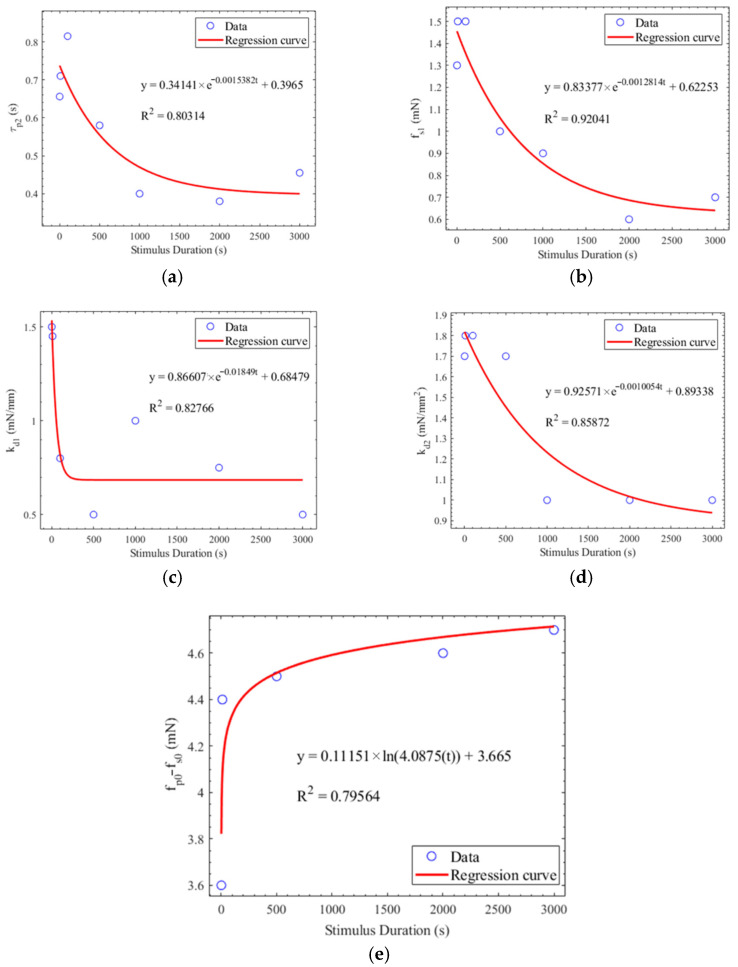
(**a**) Regression between *τ_p_*_2_ and the stimulus duration. (**b**) Regression between *f_s_*_1_ and the stimulus duration. (**c**) Regression between *k_d_*_1_ and the stimulus duration. (**d**) Regression between *k_d_*_2_ and the stimulus duration. (**e**) Regression between *f_p_*_0_-*f_s_*_0_ and the stimulus duration.

The PLR inherent model was verified by predicting the PLR response caused by different stimulus durations. The regression between the parameters and the stimulus durations in Table 2 was calculated. The coefficient of variation was applied to find which parameters were significantly affected by the stimulus duration. Coefficient of variation $C_{v}$ was written as

(6) $$C_{v}=\frac{\sigma }{\mu },$$

where C*_v_* represents the coefficient of variation, *σ* represents standard deviation, and *μ* represents the average. Table 3 shows the average, standard deviation, and coefficient of variation of the parameters under different stimulus durations. $C_{v}$ equal to 0.15 was defined as the threshold for determining whether the parameters were susceptible to the stimulus duration. The stimulus duration of 10 ms was used to predict other PLRs under the stimulus durations of 1 ms, 100 ms, 500 ms, 1 s, 2 s, and 3 s. If the C*_v_* of the parameter is less than 0.15, the parameters are unaffected by the stimulus durations. These parameters of $\tau_{p1}$, $\tau_{s1}$, $\tau_{s2}$, $f_{p0}$, and $l_{0}$, extracted from the PLR at the stimulus duration of 10 ms, were directly applied to predict the PLR under all the other stimulus durations. Figure 8 shows the regression curves of the parameters significantly affected by the stimulus durations. The value of these parameters used to predict the PLR response was decided by the regression function between these parameters and the stimulus durations.

**Figure 9:**
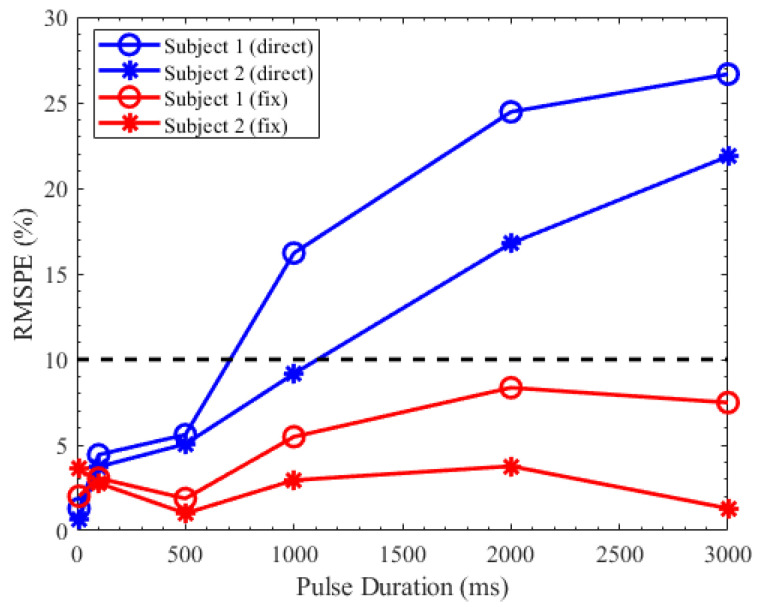
RMSPE between the measured pupillary light reflex (PLR) response and the predicted PLR response.

Figure 9 shows the RMSPE between the predicted PLR response and the measured PLR response. The blue line is the predicted PLR response that the parameters extracted from the PLR under the stimulus duration of 10 ms were directly applied to predict the PLR response under other stimulus durations. The RMSPE between the measured and predicted PLR response increased as the stimulus duration increased. The red line is the predicted PLR with which the parameters were calculated by the regression equations of these parameters. Each prediction had a similarity of more than 90%. Although the predicted PLR responses generated by our model had a slight deviation, the similarity between the predicted PLR responses and the measured PLR responses was over 90%.

### 2.5. Sensitivity Analysis of PLR Inherent Model

**Figure 10:**
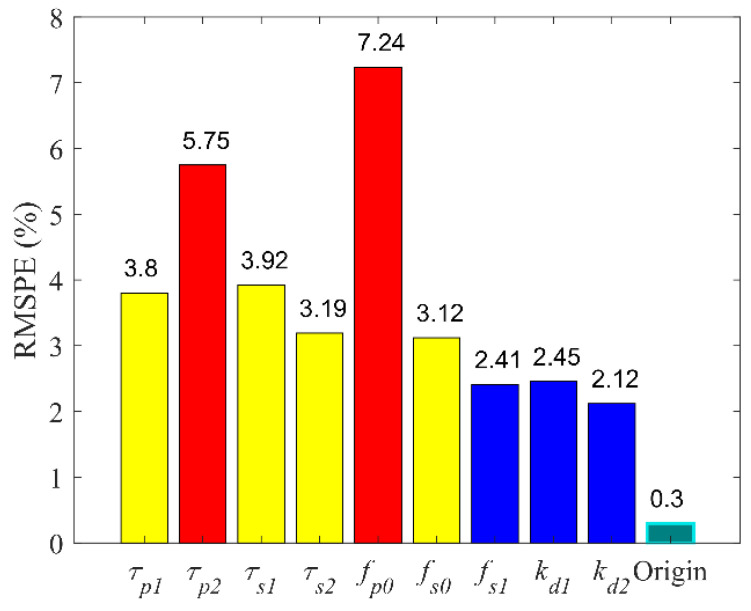
Sensitivity of nine parameters in pupillary light reflex inherent model.

Sensitivity analysis aims to study how much model output is affected by variation in model input or the model parameters. The sensitivity of the PLR inherent model complied with accuracy to the parameters listed in Table 2. One parameter was increased by 10% at a time, while the other parameter was constant, and the RMSPE between the model output and the experimental data was calculated. The RMSPE of each increasing parameter is depicted in Figure 10. The RMSPE increased by more than ten times in changing *τ*_*p*2_, *τ*_*s*1_, *τ*_*s*2_, *f*_*p*0_ and increased by 15 times in changing *f*_*p*0_ and *τ*_*p*2_. Compared to other parameters, *f*_*p*0_ and *τ*_*p*2_ significantly affected the accuracy of model simulation.

## 3. Autonomic Neural Transmission Analysis

### 3.1. Material and Experiment

A total of 84 participants, including 42 healthy people and 42 people with DM, participated in the experiment at National Taiwan University Hospital. No participants had a history of eye trauma, history of an eye operation, keratopathy, cataracts, color blind, color weakness, epilepsy, systemic diseases, retinal diseases, or high myopia. Table 4 presents the basic statistical information of participants.

The experimental procedure was the same as the previous study [24]. All participants took dark adaption in a dark room for 2 min and then underwent two tests, each including four stimuli of white, red, green, and blue light. The stimulus intensity of one test was 0.2 cd, and the other one was 1.2 cd. In all stimuli, the right eyes of all participants were simulated for 10 ms, and both eyes of all participants were recorded for 25 s. The participants were provided with a rest for 25 s between each stimulus and rest for 5 min between two tests.

### 3.2. Coherence between Direct Response and the Consensual Response of Pupillary Light Reflex

This section took the pupillary response of healthy participants under white light stimulus as an example to discuss the coherence between direct response and consensual response. The direct response was the PLR response of the stimulated right eye and the consensual response was that of the non-stimulated left eye. A total of 10 parameters of the PLR inherent model were used to observe the difference of PLR response between stimulated eyes and consensual eyes. The percentage error (7) of all parameters between both eyes was calculated. Although the parameters, *τ*_*p*1_, *τ*_*p*2_, *τ*_*s*1_, *τ*_*s*2_, and *l*_0_, exhibited a slight difference between the two responses, the percentage errors were all below 10%. Table 5 shows the results of the experiment.

(7) $$\mathrm{PE}\left(\%\right)=\frac{Parameter_{Right}-Parameter_{Left}}{Parameter_{Right}}\times 100\%.$$

### 3.3. The Coherence of Pupillary Light Reflex between Four Light Stimuli

This section took the pupillary response of the right eye in healthy participants as an example to discuss the PLR difference between four light stimuli. The coefficient of variation was performed (6). Table 6 presents the statistical results of 10 parameters in white, red, green, and blue stimuli. Consequently, the total of nine parameters exhibited a slight difference between four stimuli; the $C_{v}$ of these parameters were all below 0.1.

### 3.4. PLR Comparison between Healthy People and Those with DM

In this section, we compared the difference in autonomic neurotransmission between healthy people and those with DM by applying the parameters in PLR inherent model. Because the parameters showed neither significant differences between direct PLR response and consensual response nor significant differences between four stimuli, all PLR data of both eyes were divided into those of healthy participants and those of participants with DM regardless of stimulus intensity and stimulus color; thus, a total of 336 healthy PLR samples and 336 PLR samples, which included the PLR of both eyes under two intensities and four stimuli, was recruited in tests. Two groups coming from the same population should be examined, so a two-sample *t*-test was used:

(8) $$t=\frac{\left(\bar{X_{1}}-\bar{X_{2}}\right)-\left(\mu_{1}-\mu_{2}\right)}{\sqrt{\frac{S_{1}{}^{2}}{n_{1}}+\frac{S_{2}{}^{2}}{n_{2}}}},$$

where $\bar{X_{1}}\;\mathrm{and}\;\bar{X_{2}}$ represent the mean of two groups, $S_{1}\;\mathrm{and}\;S_{2}$ represent the standard deviation of the two groups, and $n_{1}\;\mathrm{and}\;n_{2}$ represent the sample number of two groups. Null hypothesis (*H_0_*) was applied in this test, assuming that $\mu_{1\;}-\mu_{2}=0$. *t* can be converted to *p* by *t*-distribution. If *p* is less than 0.05, the null hypothesis is rejected, which represents the two groups are significantly different. Table 7 presents the results of the tests. A total of 10 parameters, comprising *τ*_*s*1_, *f*_*s*0_, *k*_*d*1_, *l_*0*_*, *τ*_*p*2_-*τ*_*s*1_, *f_p*0*_-f_s*0*_*, *τ*_*s*1_-*τ*_*p*1_, *Imp_*1*_*, *Imp_*2*_*, and l_*0*_′ * k_d*1*_, exhibited significant differences between healthy participants and those with DM. *l_*0*_′* was *l_*0*_* divided by the diameter of the iris.

### 3.5. Receiver Operating Characteristic Analysis between Healthy People and Those with DM

This study also tried to assess the diabetic autonomic neuropathy by examining the PLR abnormalities in people with DM; thus, the ROC analysis was used to evaluate which parameters of the PLR inherent model were the best index to identify the abnormalities of the PLR between healthy people and those with DM. Consequently, the top 10 parameters listed in Table 7 did not exhibit good results, so the area under the curve (AUC) of these parameters was less than 0.6; however, we tried to create a factor from these parameters to achieve a better AUC. According to the statistical results in Table 7, *Imp_*2*_* and l_*0*_ * k_d*1*_ achieved significant differences between healthy participants and those with DM, and correlated with the other parameters that also achieved significant differences; Thus, the *DAN* factor was defined as:

(9) $$DAN=Imp_{2}*{l_{0}}^{\prime }*k_{d1}$$

**Figure 11:**
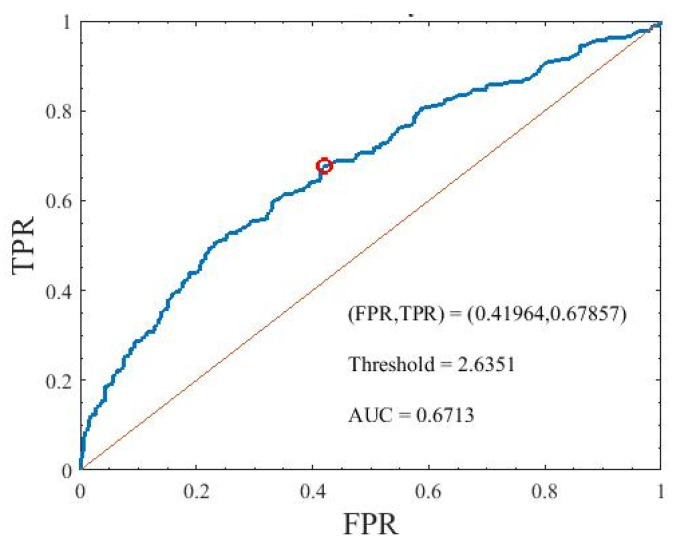
Area under the curve of the DAN factor used to identify the participants with diabetes mellitus. TPR is true positive rate and FPR is false positive rate.

Figure 11 shows the ROC curve of the DAN factor. The AUC of the factor was 0.6713. The sensitivity and specificity of that were 0.6786 and 0.5804, respectively.

## 4. Discussion

In the PLR inherent model, the first-order term of elastic force with *k*_*d*1_ was contained to improve the description of the elastic force in pupillary dilator and pupillary constrictor. The elastic force, the viscous force, and the ANS forces were assumed to be zero when the pupil was in a dark-adapted resting state; the SNS force was assumed to join the pupillary constriction before the maximum pupil constriction and still active in pupillary dilation while the force of PSNS stopped. According to the fitting result, *τ*_*p*1_ was approximately 200 ms under all stimulus durations; this result is consistent with that of Fan’s model [37], whereas *τ*_*s*1_ was not consistent with that of Fan’s because the assumption of SNS force was different. Compared to Fan’s model, our model precisely simulated the PLR response when the stimulus duration was less than 3 s (Figure 6). Moreover, the RMSEs of the predicted PLR response were less than 8.5% when the model parameters were derived from the regression by the measured PLR response.

A total of 18 parameters in the PLR inherent model were used to compare the PLR difference in people with and without DM. *τ*_*s*1_ of the participants with DM was significantly longer than those of the healthy participants (*p* = 1.44E × 10^−5^). *f*_*s*0_ and *f_p*0*_-f_s*0*_* of the participants with DM were larger and lower than those of the healthy participants (*p* = 0.384 and *p* = 0.004), respectively. *τ*_*s*1_ and f_s0_ are mainly associated with the later stage of pupillary constriction and the early stage of pupillary dilation. The increase of *τ*_*s*1_ and *f*_*s*0_ could increase the maximum pupil constriction velocities (MCV). The increased MCV of the people with DM compared to that of healthy people was also found in previous studies [24]. The *k*_*d*1_ and the *Imp*_2_ of the participants with DM were significantly smaller than those of the healthy participants (*p* = 0.081 and *p* = 4.55 × 10^−7^). The decrease in *k*_*d*1_ and *Imp_*2*_* is related to the decrease in maximum pupillary restoration velocities (MRV) and average pupillary restoration velocities. The reduced MRV in people with autonomic dysfunction or DM was also reported by Muppidi et al. [25] and the study [24]. The reduced MRV in people with DM was also found by Jain et al. [20] and the study [24]. Ishikawa et al. [39] reported that the constrictor presented normal nerve endings, but the dilator had some degenerated nerve endings; this indicates sympathetic damage.

The altered function of efferent fibers from the ciliary ganglion to the iris might be the reason for the abnormal PLR. The afferent limb of PLR is exclusively mediated by melanopsin-expressing retinal ganglion cells (mRGCs) [40]. The mRGCs not only receive synaptic input from rod and cone cells but also directly respond to light. The mRGCs convey the synaptic signal into the olivary pretectal nucleus in turn to the edinger-westphal nucleus (EWN). Afterward, the EWN innervates the constrictor by means of the ciliary ganglion neurons [41]. Kumar et al. found that the diabetic mouse had faster pupillary constriction and slower dilation than the healthy mouse under a 488-nm high intensity blue laser light [42]. These symptoms were correlated to the extensive dendritic network of the mRGCs and increased melanopsin mRNA in the diabetic mouse. Kumar et al. concluded that the pathological changes of the mRPGs during early diabetic retinopathy might contribute to the changes in PLR. However, the PLR associated with mRPGs is induced by short-wavelength (blue) and high-intensity light in longer stimulus durations [43]; this stimulus condition is different from the current study.

The inherent PLR model in the current study could not directly explain the pathological changes in people with DM but could observe the changes in pupillary constriction and dilation controlled by the sympathetic and parasympathetic nervous system under a short-pulse white light stimulus. In summary, the parameters (e.g., *τ*_*s*1_ and *f*_*s*0_) relative to SNS were all significantly different between healthy participants and those with DM. The reason could be that the sympathetic nervous system, which controls the dilator muscle, altered in diabetic patients.

## 5. Conclusions

In this study, we used the PLR inherent model to explore the PLR abnormalities in people with DM. By extracting the parameters from the participants with and without DM, the abnormalities relating to ANS could be observed; according to the statistical test results in Table 7, the dilator and the nerve endings of SNS might degenerate in people with DM whose diabetic duration is less than ten years. However, one of our limitations was that the results were not further verified by directly observing the ANS of people with and without DM. In the future, the number of samples of the experiments could be increased and to explore the abnormities of the ANS in people with DM by microscope or ultrasonic equipment to verify the testing results of the PLR.

## Figures and Tables

**Table 1:** Initial value and boundary value of the parameter for model simulation and prediction.

Parameter	Initial Value	Unit	Lower Bound	Upper Bound	Step
$\tau_{p1}$	0.250	s	0.150	0.300	0.005
$\tau_{p2}$	1.250	s	0.200	1.250	0.005
$\tau_{s1}$	0.500	s	0.270	0.650	0.005
$\tau_{s2}$	1.300	s	1.200	2.000	0.005
$f_{p0}$	11.00	mN	8.000	14.000	0.100
$f_{s0}$	7.000	mN	4.000	15.000	0.100
	1.000	mN	0.100	1.500	0.100
D	4.300	g/s	…	…	…
$k_{d1}$	1.000	mN/mm	0.500	1.500	0.050
$k_{d2}$	1.600	mN/mm^2^	1.000	1.800	0.050

**Table 2:** Extracting parameters of the measured pupillary light reflex in seven stimulus durations.

Parameter	1 ms	10 ms	100 ms	500 ms	1 s	2 s	3 s
$\tau_{p1}$ (s)	0.235	0.205	0.175	0.220	0.215	0.165	0.210
$\tau_{p2}$ (s)	0.656	0.710	0.815	0.580	0.400	0.380	0.455
$\tau_{s1}$ (s)	0.415	0.425	0.545	0.565	0.555	0.570	0.570
$\tau_{s2}$ (s)	1.520	1.205	1.215	1.365	1.395	1.545	1.370
$f_{p0}\;$(mN)	10.900	11.1000	12.20	11.300	11.100	10.800	11.300
$f_{s0}$ (mN)	7.300	6.700	9.700	6.800	5.800	6.200	6.600
$f_{s1}$ (mN)	1.300	1.500	1.500	1.000	0.900	0.600	0.700
$k_{d1\;}$($\frac{mN}{mm}$)	1.500	1.450	0.800	0.500	1.000	0.750	0.500
$k_{d2\;}$($\frac{mN}{mm^{2}}$)	1.700	1.800	1.800	1.700	1.000	1.000	1.000
D (g/s)	4.300	4.300	4.300	4.300	4.300	4.300	4.300

**Table 3:** Coefficient of variation of parameters.

Parameter	$\tau_{p1}$	$\tau_{p2}$	$\tau_{s1}$	$\tau_{s2}$	$f_{p0}$	$f_{s0}$	$f_{s1}$	$k_{d1}$	$k_{d2}$	$l_{0}$
σ	0.204	0.571	0.521	1.374	11.243	7.014	1.071	0.929	1.429	3.245
μ	0.025	0.166	0.069	0.132	0.461	1.275	0.368	0.412	0.403	0.029
$C_{v}$	0.123	0.291	0.133	0.096	0.041	0.182	0.344	0.444	0.282	0.009

**Table 4:** Basic characteristics of the participants.

	Healthy Participants	Participants with DM
Male: Female	26/16	29/13
Age (years old)	37 ± 17	47 ± 14
Original HbA1c (years old)	<6	8.9 ± 2.9
Current HbA1c (years old)	<6	7.6 ± 2.1
Duration (years old)	…	7.1 ± 7
Diabetic type	…	Type I and 2

**Table 5:** Percentage error of 10 parameters between stimulated eye (right eye) and non-stimulated eye (left eye) in two participants.

Parameters	Healthy Participant 1			Healthy Participant 2		
	Right	Left	PE (%)	Right	Left	PE (%)
*τ*_*p*1_ *	0.19	0.200	5.263	0.230	0.245	6.522
*τ*_*p*2_ *	0.475	0.500	5.263	0.595	0.630	5.882
*τ*_*s*1_ *	0.360	0.375	4.167	0.395	0.420	6.329
*τ*_*s*2_	1.215	1.210	−0.412	1.195	1.195	0.000
*f*_*p*0_	11.400	11.300	−0.877	10.800	10.700	−0.926
*f*_*s*0_ *	5.000	5.300	6.000	6.600	7.100	7.576
*f*_*s*1_	1.500	1.500	0.000	1.500	1.500	0.000
*k*_*d*1_	1.500	1.500	0.000	1.500	1.500	0.000
*k*_*d*2_	1.800	1.800	0.000	1.800	1.800	0.000
*l_*0*_*	2.534	2.681	5.773	2.706	2.784	2.89

* indicates that percentage error (PE) is larger than 2%.

**Table 6:** Model parameters of measured pupillary response in different stimulus durations.

Parameter	W	R	G	B	Mean	Std	C*_v_*
*τ*_*p*1_	0.224	0.201	0.197	0.190	0.203	0.015	0.0725
*τ*_*p*2_	0.716	0.787	0.795	0.807	0.776	0.041	0.0528
*τ*_*s*1_	0.416	0.418	0.428	0.423	0.421	0.005	0.0128
*τ*_*s*2_	1.375	1.420	1.358	1.403	1.389	0.028	0.0200
*f*_*p*0_	10.036	10.564	10.671	10.707	10.495	0.312	0.0297
*f*_*s*0_	7.536	7.876	7.831	7.598	7.71	0.168	0.0218
*f*_*s*1_	1.319	1.224	1.281	1.226	1.263	0.046	0.0364
*k*_*d*1_	1.277	1.292	1.260	1.224	1.263	0.029	0.0232
*k*_*d*2_	1.657	1.635	1.690	1.664	1.662	0.023	0.0136
*l_*0*_*	3.098	3.070	3.038	2.991	3.049	0.046	0.0151

**Table 7:** Statistical results of the parameters between participants with and without diabetes mellitus.

Parameters	Healthy Participants (336 Samples)		Participants with DM (336 Samples)		*p*-Value	
	**Mean**	**STD**	**Mean**	**STD**		
*τ*_*p*1_	0.205	0.035	0.208	0.038	0.2842	
*τ*_*p*2_	0.779	0.142	0.772	0.14	0.5357	
*τ*_*s*1_	0.423	0.045	0.439	0.05	$1.44\times 10^{-5}$	**
*τ*_*s*2_	1.391	0.228	1.397	0.24	0.7035	
*f*_*p*0_	10.573	1.193	10.549	1.189	0.7881	
*f*_*s*0_	7.745	1.547	7.991	1.525	0.0384	*
*f*_*s*1_	1.264	0.304	1.279	0.282	0.4946	
*k*_*d*1_	1.251	0.257	1.195	0.286	0.0081	*
*k*_*d*2_	1.656	0.217	1.676	0.208	0.2276	
*l_*0*_*′	3.051	0.392	2.783	0.381	$2.41\times 10^{-18}$	**
*τ*_*p*2_-*τ*_*s*1_	0.356	0.131	0.333	0.129	0.0240	*
*f_p*0*_-f_s*0*_*	2.828	1.21	2.558	1.258	0.0046	*
*τ*_*s*1_-*τ*_*p*1_	0.219	0.053	0.232	0.066	0.0051	*
*τ*_*s*2_-*τ*_*p*2_	0.611	0.231	0.625	0.229	0.4436	
*Imp_*1*_*	2.333	0.723	2.481	0.844	0.0150	*
*Imp_*2*_*	0.964	0.481	0.79	0.401	$4.55\times 10^{-7}$	**
*Imp_*3*_*	0.749	0.266	0.766	0.247	0.3904	
l_0_′ * k_d*1*_	3.799	0.846	3.305	0.835	$8.69\times 10^{-14}$	**
*τ*_*p*1_	0.205	0.035	0.208	0.038	0.2842	

* indicates that *p* < 0.05 and ** indicates that *p* < 0.001.

## Data Availability

The data presented in this study are available on request from the corresponding author. The data are not publicly available due to their containing information that could compromise the privacy of research participants and the IRB statement (103-054-E).
